# Supplementary material for: YouTube as a Source of Patient Information for Meningiomas: A Content Quality and Audience Engagement Analysis
Source: Healthcare (Basel). 2022 Mar 10;10(3):506. doi: 10.3390/healthcare10030506 (PMC8953264; doi:10.3390/healthcare10030506)
Supplement: Supplementary file 1 [file healthcare-10-00506-s001.zip › healthcare-1610009-supplementary.pdf]

Supplementary Table S1.

		Indications for surgery		Treatment options		Localization and clinical manifestations		Treatment risks		Follow up		Adjuvant treatment		Results of treatment/prognosis		Diagrams		Doctor speaker	
		**With**	**Without**	**With**	**Without**	**With**	**Without**	**With**	**Without**	**With**	**Without**	**With**	**Without**	**With**	**Without**	**With**	**Without**	**With**	**Without**
**DISCERN**	**Count**	31,00	88,00	84,00	35,00	41,00	78,00	15,00	104,00	20,00	99,00	25,00	94,00	34,00	85,00	16,00	103,00	92,00	27,00
	**Mean**	43,91	32,63	38,30	29,02	42,66	31,84	46,75	33,96	46,17	33,43	47,47	32,40	43,84	32,26	44,73	34,15	37,34	29,54
	**Median**	42,80	30,80	37,10	28,00	41,20	30,80	44,80	32,20	45,90	31,60	45,80	30,80	44,90	30,80	44,30	31,80	35,50	28,60
	**Standard deviation**	9,44	7,15	9,17	5,25	9,31	6,67	8,84	8,12	7,90	7,92	7,34	6,76	9,29	6,84	8,46	8,52	9,21	6,34
	**-95% confidence interval**	40,45	31,12	36,31	27,22	39,72	30,34	41,85	32,38	42,47	31,85	44,44	31,02	40,59	30,79	40,21	32,48	35,43	27,03
	**+95% confidence interval**	47,37	34,14	40,28	30,83	45,60	33,34	51,64	35,54	49,87	35,01	50,50	33,79	47,08	33,74	49,24	35,81	39,24	32,05
	**p**	0,000		0,000		0,000		0,000		0,000		0,000		0,000		0,000		0,000	
**GQS**	**Count**	31,00	88,00	84,00	35,00	41,00	78,00	15,00	104,00	20,00	99,00	25,00	94,00	34,00	85,00	16,00	103,00	92,00	27,00
	**Mean**	2,87	2,29	2,59	2,09	2,85	2,22	3,00	2,36	2,95	2,34	3,09	2,27	2,94	2,24	2,78	2,39	2,54	2,09
	**Median**	2,80	2,30	2,60	2,00	2,80	2,20	3,20	2,40	2,90	2,40	3,20	2,20	3,00	2,20	2,60	2,40	2,50	2,20
	**Standard deviation**	0,67	0,61	0,66	0,60	0,59	0,62	0,71	0,64	0,55	0,66	0,61	0,59	0,57	0,61	0,64	0,67	0,67	0,59
	**-95% confidence interval**	2,62	2,16	2,45	1,88	2,67	2,08	2,61	2,24	2,69	2,21	2,84	2,15	2,74	2,11	2,43	2,26	2,41	1,85
	**+95% confidence interval**	3,12	2,42	2,73	2,29	3,04	2,36	3,39	2,48	3,21	2,47	3,34	2,39	3,13	2,37	3,12	2,52	2,68	2,32
	**p**	0,000		0,000		0,000		0,003		0,000		0,000		0,000		0,049		0,004	
**JAMA**	**Count**	31,00	88,00	84,00	35,00	41,00	78,00	15,00	104,00	20,00	99,00	25,00	94,00	34,00	85,00	16,00	103,00	92,00	27,00
	**Mean**	2,12	1,67	1,93	1,46	2,01	1,67	2,17	1,73	2,28	1,69	2,18	1,68	2,10	1,66	2,39	1,69	1,93	1,31
	**Median**	2,00	1,50	1,88	1,50	2,00	1,50	2,00	1,50	2,00	1,50	2,00	1,50	2,00	1,50	2,38	1,50	1,75	1,00
	**Standard deviation**	0,63	0,69	0,74	0,47	0,68	0,68	0,67	0,69	0,74	0,65	0,74	0,65	0,76	0,64	0,54	0,68	0,68	0,54
	**-95% confidence interval**	1,89	1,52	1,77	1,30	1,80	1,52	1,79	1,60	1,93	1,56	1,87	1,55	1,83	1,53	2,10	1,56	1,78	1,10
	**+95% confidence interval**	2,35	1,82	2,09	1,62	2,23	1,82	2,54	1,87	2,62	1,82	2,49	1,82	2,36	1,80	2,68	1,83	2,07	1,53
	**p**	0,000		0,001		0,003		0,012		0,000		0,001		0,000		0,000		0,000	

Supplementary Table S2.

		Symptoms		Predisposing factors		Epidemiology		Clean information		When to seek medical attention	
		With	Without	With	Without	With	Without	With	Without	With	Without
DISCERN	Count	88,00	31,00	39,00	80,00	41,00	78,00	67,00	52,00	7,00	112,00
	Mean	37,22	30,87	41,95	32,46	41,12	32,65	38,14	32,26	48,26	34,78
	Median	35,60	29,20	41,20	31,10	41,20	31,60	36,20	31,60	45,60	33,20
	Standard deviation	9,39	6,92	10,10	6,93	11,05	6,47	10,20	6,50	9,65	8,63
	-95% confidence interval	35,23	28,33	38,68	30,91	37,63	31,19	35,65	30,45	39,33	33,16
	+95% confidence interval	39,21	33,41	45,23	34,00	44,61	34,11	40,62	34,07	57,18	36,39
	p	0,001		0,000		0,000		0,002		0,001	
GQS	Count	88,00	31,00	39,00	80,00	41,00	78,00	67,00	52,00	7,00	112,00
	Mean	2,54	2,17	2,93	2,20	2,76	2,27	2,64	2,18	3,34	2,38
	Median	2,60	2,20	3,00	2,20	2,80	2,40	2,60	2,20	3,20	2,40
	Standard deviation	0,64	0,73	0,64	0,56	0,77	0,56	0,69	0,57	0,41	0,65
	-95% confidence interval	2,40	1,90	2,73	2,08	2,51	2,15	2,47	2,03	2,96	2,26
	+95% confidence interval	2,67	2,43	3,14	2,32	3,00	2,40	2,81	2,34	3,72	2,51
	p	0,013		0,000		0,000		0,000		0,000	

Supplementary Table S3.

		Genetical predisposition		How to perform procedure		Animation		Radiological data		Misleading info	
		With	Without	With	Without	With	Without	With	Without	With	Without
JAMA	Count	15,00	104,00	28,00	91,00	40,00	79,00	86,00	33,00	35,00	84,00
	Mean	2,08	1,75	2,25	1,65	2,04	1,66	1,87	1,58	1,54	1,89
	Median	2,00	1,50	2,00	1,50	2,00	1,50	1,75	1,50	1,50	1,75
	Standard deviation	0,69	0,69	0,79	0,61	0,75	0,64	0,72	0,60	0,60	0,72
	-95% confidence interval	1,70	1,61	1,94	1,52	1,80	1,52	1,71	1,36	1,34	1,73
	+95% confidence interval	2,46	1,88	2,56	1,77	2,28	1,81	2,02	1,79	1,75	2,05
	p	0,046		0,000		0,004		0,031			0,007

		Histology		Embolization		WHO classification	
		With	Without	With	Without	With	Without
DISCERN	Count	19,00	100,00	11,00	108,00	15,00	104,00
	Mean	32,60	36,13	42,87	34,82	42,51	34,57
	Median	28,00	35,40	40,40	32,80	42,80	33,50
	Standard deviation	11,52	8,67	8,63	8,99	13,23	8,10
	-95% confidence interval	27,05	34,41	37,07	33,11	35,18	32,99
	+95% confidence interval	38,15	37,85	48,67	36,54	49,83	36,14
	p	0,009		0,004		0,031	

Supplementary Table S4. Audience engagement

		Animations	
		**With**	**Without**
**Views**	**Count**	40,00	79,00
	**Mean**	47205,43	12567,46
	**Median**	10568,00	3910,00
	**Standard deviation**	110909,16	21559,60
	**- 95% confidence interval**	11734,95	7738,37
	**+ 95% confidence interval**	82675,90	17396,54
	**p**	0,005	
**Likes**	**Count**	40,00	79,00
	**Mean**	386,50	142,61
	**Median**	65,50	35,00
	**Standard deviation**	1000,83	369,47
	**- 95% confidence interval**	66,42	59,85
	**+ 95% confidence interval**	706,58	225,36
	**P**	0,002	
**Dislikes**	**Count**	40,00	79,00
	**Mean**	16,60	4,09
	**Median**	3,00	1,00
	**Standard deviation**	40,75	7,43
	**- 95% confidence interval**	3,57	2,42
	**+ 95% confidence interval**	29,63	5,75
	**P**	0,009	
**Comments**	**Count**	35,00	72,00
	**Mean**	57,26	11,90
	**Median**	8,00	3,00
	**Standard deviation**	163,51	21,69
	**- 95% confidence interval**	1,09	6,81
	**+ 95% confidence interval**	113,42	17,00
	**P**	0,010	
**Subscribers**	**Count**	40,00	76,00
	**Mean**	64389,00	48435,59

	**Median**	45200,00	19050,00
	**Standard deviation**	98999,67	82195,36
	**- 95% confidence interval**	32727,37	29653,15
	**+ 95% confidence interval**	96050,63	67218,04
	**P**	0,036	
**VPI**	**Count**	40,00	79,00
	**Mean**	32,85	7,98
	**Median**	5,65	3,16
	**Standard deviation**	90,48	16,87
	**- 95% confidence interval**	3,91	4,20
	**+ 95% confidence interval**	61,79	11,75
	**P**	0,002	

		Misinformation	
**Like ratio**	**Count**	35,00	84,00
	**Mean**	79,06	93,88
	**Median**	95,18	97,01
	**Standard deviation**	36,80	15,45
	**- 95% confidence interval**	66,41	90,53
	**+ 95% confidence interval**	91,70	97,23
	**P**	0,036	

Supplementary Table S5. Top 5 DISCERN videos.

	**Mean DISCERN score**	**VPI score**	**Mean GQS score**	**Mean JAMA score**	**Symptoms**	**Definition**	**Risk factors**	**Pregnancy and meningiomas**	**WHO classification**	**Epidemiology**	**Misinformation**	**Who was the speaker**	**Country**	**Time (s)**	**Like ratio**
**1**	62,00	29,68	3,60	2,25	+	+	+	-	+	+	+	Doctor	USA	3172	96,95
**2**	57,00	22,73	4,00	3,00	+	+	+	-	+	+	+	Doctor	USA	3651	94,07
**3**	56,60	5,28	4,00	2,50	+	+	+	+	+	+	-	Doctor	-	2768	95,56
**4**	55,60	0,44	3,20	2,00	+	-	+	-	+	+	-	Doctor	-	2734	80,00
**5**	54,20	5,48	3,60	2,50	+	-	+	-	-	+	+	Doctor	USA	3523	93,78

Supplementary Table S6. TOP 5 VPI videos

	VPI score	Mean DISCERN score	Mean GQS score	Mean JAMA score	Symptoms	Definition	Risk factors	Pregnancy and meningiomas	WHO classification	Epidemiology	Misinformation	Speaker	Country	Time (s)	Like ratio
1	510,63	28,80	2,40	1,00	-	-	-	-	-	-	-	Doctor	-	513	96,34
2	278,52	30,60	2,00	1,50	+	-	-	-	-	-	+	Doctor	-	350	94,61
3	135,26	25,40	2,60	1,25	-	+	-	-	-	-	-	-	USA	1190	98,46
4	102,00	24,20	1,80	1,00	+	-	-	-	-	-	-	-	USA	119	96,57
5	73,33	37,00	2,40	2,50	+	-	-	-	-	-	-	Doctor	-	268	94,52
